# Improvement of Performance, Stability and Continuity by Modified Size-Consistent Multipartitioning Quantum Mechanical/Molecular Mechanical Method

**DOI:** 10.3390/molecules23081882

**Published:** 2018-07-27

**Authors:** Hiroshi C. Watanabe

**Affiliations:** 1 Quantum Computing Center, Keio University, 3-14-1 Hiyoshi, Kohoku-ku, Kawasaki 223-8522, Japan; hcwatanabe@keio.jp; Tel.: +81-45-566-1817; 2 Graduate School of Science and Technology, Keio University, 3-14-1 Hiyoshi, Kohoku-ku, Kawasaki 223-8522, Japan; 3 Japan Science and Technology Agency, PRESTO 4-1-8, Honcho, Kawaguchi, Saitama 332-0012, Japan

**Keywords:** quantum mechanics/molecular mechanics, molecular dynamics, adaptive QM/MM, condensed matter, solvation

## Abstract

For condensed systems, the incorporation of quantum chemical solvent effects into molecular dynamics simulations has been a major concern. To this end, quantum mechanical/molecular mechanical (QM/MM) techniques are popular and powerful options to treat gigantic systems. However, they cannot be directly applied because of temporal and spatial discontinuity problems. To overcome these problems, in a previous study, we proposed a corrective QM/MM method, size-consistent multipartitioning (SCMP) QM/MM and successfully demonstrated that, using SCMP, it is possible to perform stable molecular dynamics simulations by effectively taking into account solvent quantum chemical effects. The SCMP method is characterized by two original features: size-consistency of a QM region among all QM/MM partitioning and partitioning update. However, in our previous study, the performance was not fully elicited compared to the theoretical upper bound and the optimal partitioning update protocol and parameters were not fully verified. To elicit the potential performance, in the present study, we simplified the theoretical framework and modified the partitioning protocol.

## 1. Introduction

In molecular simulations, the choice of molecular models has been a major concern and always a product of compromise between accuracy and computational cost. To optimize this balance, multiscale simulations, which combine more than two molecular models, have been proposed by many research groups. Among the various multiscale techniques, the quantum-mechanical (QM)/molecular-mechanical (MM) method has been the most popular and successful one. While QM methods are transferable and accurate because they explicitly treat the electronic structure, they require massive computation time and resources. Therefore, they have severe limitations regarding the size of the systems that can be treated and the simulation times that can be achieved. In the contrast, the QM/MM method makes partial electronic structures of gigantic systems available with moderate computational load. Thus, it has been widely applied to investigate reactions in biomolecules and solutions. To apply QM/MM methods within a molecular dynamics (MD) simulation framework, there are two possible options. The first one is to define only the solute molecule as QM and the rest as MM. The second option is to expand the QM region to the surrounding solvent molecules. Apparently and intuitively, the latter option seems to yield a more reasonable and natural picture of the molecular structure and dynamics because of the incorporation of environmental quantum chemical effects. Regarding dynamics simulations, however, mobile solvent molecules diffuse away from the QM solute in course of time. As a result, the solvent quantum effect is lost, although the computational cost remains substantially larger than in the former setting, which treats all solvent molecules as MM. Therefore, in conventional QM/MM MD simulations, quantum chemical solvent effects cannot be taken into consideration.

To overcome this limitation, various corrective approaches have been proposed by several research groups in the last two decades [1,2,3,4,5,6,7,8,9,10,11]. These approaches are categorized into two groups: “constrained” and “adaptive” QM/MM methods. The constrained methods, such as “flexible inner region ensemble separator” (FIRES) [1], “boundary based on exchange symmetry theory” (BEST) [2] and “boundary constraint with correction” (BCC) [3], employ constraints on QM (and MM) solvent molecules to prevent their diffusion. These methods are relatively stable, in particular the Hamiltonian is well conserved in BCC. In addition, their computational cost is moderate compared to that of the methods belonging to the other group because a particular division of the molecular system into QM and MM regions, which is termed a QM/MM partitioning, is fixed during the simulation. Since the “constrained” methods do not allow the exchange of solvent molecules through the QM/MM border, their dynamics is not realistic. Instead, they are designed to investigate solvation structures (e.g., through the calculation of radial distribution functions) using ensemble averages. These methods are controversial because they assume that ensemble averages are rigorously accurate only if there is a symmetric exchange between QM and MM solvent molecules; that is, they suppose that QM and MM solvent molecules are energetically identical.

In contrast to the constrained methods, adaptive QM/MM methods employ a flexible molecular definition and allow exchanges between QM and MM molecules. To this end, most adaptive QM/MM methods exploit a multipartitioning approach, which considers various QM/MM partitionings whose QM regions consist of different sets of solvent molecules. Based on the respective partitionings, potential energies and forces are individually calculated and then the effective energy and forces, which are used to update the coordinates along the MD simulation, are obtained as a weighted average. The adaptive QM/MM methods, such as ONIOM-XS [5], buffered-force [6], permuted adaptive partitioning (PAP) [7], modified PAP (mPAP) [8], sorted adaptive partitioning (SAP) [7], difference-based adaptive solvation (DAS) [9], scaled interaction single partition adaptive (SISPA) [10] and size-consistent multipartitioning (SCMP) [11], vary in the definition of the partitioning scheme and weight function. For comparison, most constrained and adaptive methods are also termed “single-partitioning” and “multipartitioning” methods, respectively. Since the computational cost to calculate energy and forces depends on the number of QM/MM partitionings, adaptive QM/MM methods require substantial computational resources except in the case of SISPA, which is based on a single-partitioning approach.

In the adaptive QM/MM methods, the stability of the simulations is a major concern. To have stable simulations, an adequate choice of weight function is key and the conservation of the Hamiltonian is an important index. Because of solvent diffusion, in adaptive methods, partitionings have to be redefined to keep the solute of interest surrounded by QM solvent molecules, although the abrupt update of partitionings can cause a discontinuity in the potential energy surface. Thus, a weight function is introduced to silence contributions from updated partitionings. However, in some adaptive methods, the weight function cannot completely remove the aforementioned discontinuity (denoted as temporal discontinuity), although the weight function is differentiable. In ONIOM-XS, two QM partitionings are considered, where one partitioning has a smaller QM region that does not include the solvent in transition region, while the other partitioning has a larger QM region that include them [5]. For instance, if a solvent molecule moves across the border, the forces acting on the solvent molecules in the inner layer become discontinuous, because the weight function cannot remove the contribution from the partitioning with smaller QM region, which abruptly changes the number of QM solvent molecules. In the SAP and DAS methods, temporal discontinuities appear, when the solvent number in the active zone (QM region) changes. For instance, when a solvent molecule moves from the active zone to the transition zone, an additional partitioning seems to abruptly appear with non-zero weight [7,9]. The PAP method theoretically realizes perfect continuity by taking into account all possible partitionings. However, it requires massive computation and thus is not feasible in reality [7]. To reduce the computational cost in PAP, modified PAP (mPAP) truncates the series expansion at around fifth order, although it faces the temporal discontinuity problem again [8]. In many cases, these deficiency in the weight functions result in drastic temperature drifts under microcanonical (*NVE*) conditions, as reviewed by Bulo et al. [12]. To keep a constant temperature and stabilize the MD simulations, a system may be linked to a thermostat (*NVT* ensemble). However, if the Hamiltonian is not conserved, the MD simulation does not yield accurate *NVT* ensemble. In other words, averaged observables are not physically accurate even though the temperature is moderately controlled. We proposed the size-consistent multipartitioning (SCMP) QM/MM method in 2014 [11], which numerically conserve Hamiltonian and lead to stable MD simulations over hundreds of pico seconds.

Because the force and energy calculations for each partitioning are independent in multipartitioning methods, adaptive QM/MM methods can be trivially parallelized. However, most of them are based on multiscale multipartitioning, in which the size of the QM regions varies among partitionings. Therefore, the computational loads due to the energy and force calculations are inhomogeneous among the respective partitioning, which causes idle time at several computational nodes. In contrast, all partitionings in the SCMP method have the same number of QM solvent molecules; thus, the SCMP method has high affinity with parallel computing. 

As practical applications, we have employed the SCMP methods to evaluate physicochemical properties of water and monoatomic cation solutions [13,14]. Then, we demonstrated that quantum chemical solvent effects exert a great impact on the solvation structure and dynamics of these systems by evaluating their radial distribution function, infrared spectrum and diffusion coefficient.

Although the SCMP method has shown high performance and potential, it still has vulnerability because of the weight functions, where all the weights can be zero for certain situations, and, as a result, MD simulations can crash. Furthermore, in previous studies, we also confirmed that the SCMP can connect the QM and MM regions continuously on time average but the spatial continuity is not necessarily ensured at every MD time step. Moreover, the importance of the instantaneous spatial continuity has not been discussed yet. Additionally, although the SCMP method theoretically should keep its MD performance even for highly parallelized computing, the potential was not fully elicited in a previous study [14]. Thus, in the present study, we propose modification of the SCMP theoretical framework to increase MD performance, simulation stability and spatial continuity. Then, we carry out benchmark simulations for pure water and demonstrate the updated performance.

## 2. Theory

### 2.1. Size-Consistent Partitioning

**Scheme 1:**
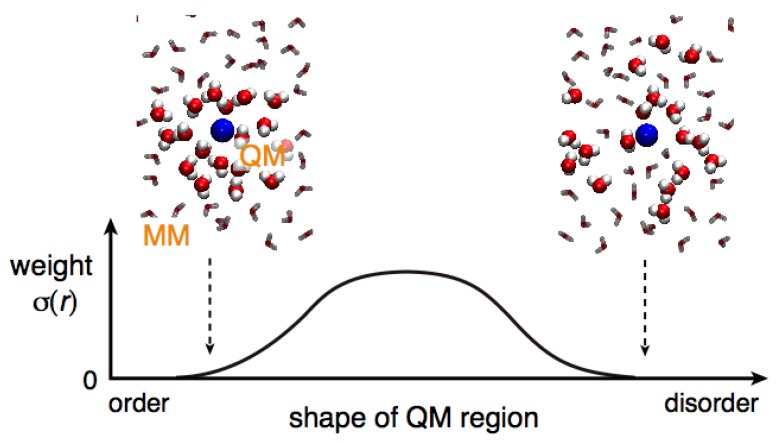
Concept of weight function. The horizontal axis represents a conceptual index related to the degree of disorder of the QM region.

Let us consider a solution system that consists of a solute of interest and *M* solvent molecules. Then, suppose a QM/MM partitioning where the QM region consists of a solute and *m* solvent molecules. Likewise, different QM/MM partitionings can be defined so that they share the same solute molecule as QM and have consistently *m* solvent molecules in the QM region but with different sets of solvent molecules. The number of the possible QM/MM partitionings is C_(*M*,*m*)_ and thus, it is not feasible to computationally cover all of them. To reduce the number of partitionings to be considered, we introduce a weight function *σ* that satisfies the following boundary conditions: *σ* = 0 in the limit of ordered and disordered partitionings as shown in Scheme 1. Here, we define a partitioning as ordered if all the nearest *m* solvent molecules to the QM solute are QM. Since the QM solvent molecules diffuse away from the solute in a QM/MM partitioning, the QM region is supposed to be disordered in the course of the MD simulation. 

If a partitioning has a perfectly ordered QM region at a given MD simulation time step, it should have a weight of zero and no contribution to the dynamics. Because of the solvent diffusion, the weight of the partitioning becomes greater than zero as the simulation evolves and then, the weight gradually goes back to zero again when the QM region is completely disordered. Because of the aforementioned conditions, a substantial number of partitionings have a weight of zero and are screened out. Although countless partitionings can still have nonzero weight, the size-consistent partitioning allows to take into account only a finite number *N* of partitionings to obtain the effective energy and force on each atom.

The SCMP method is supposed to be parallelized via Message Passing Interface (MPI), where the energy and force calculations of each QM/MM partitioning are assigned to a single node. Thus, a QM/MM partitioning having a completely disordered QM region is replaced by another one having a completely ordered QM region at the same time step. Since both completely ordered and disordered partitionings are zero-weighted, the partitioning updates do not cause any discontinuity in the effective energy and forces along the MD simulation.

### 2.2. Score Function

To numerically describe the condition *σ*^(*n*)^ mentioned in Section 2.1, we define a score function for a solvent molecule *j* in the *n*-th partitioning as *Λ_j_*^(*n*)^. According to the molecular definition, this score function varies as follows:

(1) $$\Lambda_{j}^{\left(n\right)}=\{\begin{array}{cc} \lambda_{j,\mathrm{QM}}^{\left(n\right)}\left(r_{j}\right) & \mathrm{if}\;j\in S_{\mathrm{QM}}^{\left(n\right)}\\ \lambda_{j,\mathrm{MM}}^{\left(n\right)}\left(r_{j}\right) & \mathrm{if}\;j\notin S_{\mathrm{QM}}^{\left(n\right)} \end{array}$$

where a subgroup *S*^(*n*)^_QM_ contains the QM solvent molecules in the *n*-th partitioning and *r_j_* is the distance between the QM solute and the nearest *j*-th solvent molecule; *λ*_QM_(*r_j_*) is a score function for a QM solvent, which are parameterized by range parameters *s*_QM_ and *t*_QM_ (*λ*_QM_(*r_j_*) = 1 if *r_j_* ≤ *s*_QM_ and *λ*_QM_(*r_j_*) = 0 if *t*_QM_ ≤ *r_j_*). Likewise, *λ*_MM_(*r_j_*) is a score function for an MM atom using *s*_MM_ and *t*_MM_ (*λ*_MM_(*r_j_*) = 0 for *r_j_* ≤ *s*_MM_ and *λ*_MM_(*r_j_*) = 1 for *t*_MM_ ≤ *r_j_*). In the present study, we employed spline curves for *λ*_QM_ so that

(2) $$\lambda_{\mathrm{QM}}\left(s\right)=1,\;\lambda_{\mathrm{QM}}\left(t\right)=0,\;{\frac{\partial \lambda_{\mathrm{QM}}}{\partial r}|}_{s,t=0}=0,\;{\frac{\partial^{2}\lambda_{\mathrm{QM}}}{\partial r^{2}}|}_{s,t=0}=0.$$

Also, *λ*_MM_ satisfies

(3) $$\lambda_{\mathrm{MM}}\left(s\right)=0,\;\lambda_{\mathrm{MM}}\left(t\right)=1,\;{\frac{\partial \lambda_{\mathrm{MM}}}{\partial r}|}_{s,t=0}=0,\;{\frac{\partial^{2}\lambda_{\mathrm{MM}}}{\partial r^{2}}|}_{s,t=0}=0.$$

### 2.3. Fade-in and Fade-out Functions

Using the score function introduced in Section 2.2, the fade-out function *O*^(*n*)^_QM_ is defined for the QM solvent molecules in the *n*-th partitioning as follows:

(4) $$O_{\mathrm{QM}}^{\left(n\right)}=\underset{j\in S_{\mathrm{QM}}^{\left(n\right)}}{\prod }\Lambda_{j}^{\left(n\right)}\left(r_{j}\right)$$

Note that *O*^(*n*)^_QM_ = 0 if any QM solvent molecules in the *n*-th partitioning diffuses far away (beyond *s*_QM_) from the QM solute. Otherwise, *O*^(*n*)^_QM_ > 0. Next, we define the fade-in function *I*^(*n*)^ for the QM solvent molecules in the *n*-th partitioning as follows:

(5) $$I_{\mathrm{QM}}^{\left(n\right)}=1-\underset{j\in S_{\mathrm{QM}}^{\left(n\right)}}{\prod }\Lambda_{j}^{\left(n\right)}\left(r_{j}\right)$$

*I*^(*n*)^_QM_ = 0 if all the QM solvent molecules in the *n*-th partitioning are within *s*_QM_ from the QM solute. Otherwise, *I*^(*n*)^_QM_ > 0.

Likewise, we also introduce the fade-out function *O*^(*n*)^_MM_ and the fade-in function *I*^(*n*)^_MM_ for the MM solvent molecules in the *n*-th partitioning, respectively

(6) $$O_{\mathrm{MM}}^{\left(n\right)}=\underset{j\notin S_{\mathrm{QM}}^{\left(n\right)}}{\prod }\Lambda_{j}^{\left(n\right)}\left(r_{j}\right)$$

(7) $$I_{\mathrm{MM}}^{\left(n\right)}=1-\underset{j\notin S_{\mathrm{QM}}^{\left(n\right)}}{\prod }\Lambda_{j}^{\left(n\right)}\left(r_{j}\right)$$

In contrast to the functions defined for the QM part, *O*^(*n*)^_MM_ = 0 if any MM solvent molecules in the *n*-th partitioning come within *s*_MM_ of the QM solute. Otherwise, *O*^(*n*)^_QM_ > 0. On the other hand, *I*^(*n*)^_MM_ = 0 if all the MM solvent molecules in the *n*-th partitioning are far away (beyond *t*_MM_) from the QM solute. Otherwise, *I*^(*n*)^_MM_ > 0. 

### 2.4. Weight Functions, Effective Energy and Forces

Using the fade-in and fade-out functions, the SCMP weight function of the *n*-th partitioning, *σ*^(*n*)^, can be written as:

(8) $$\sigma^{\left(n\right)}=\frac{O_{\mathrm{QM}}^{\left(n\right)}I_{\mathrm{QM}}^{\left(n\right)}O_{\mathrm{MM}}^{\left(n\right)}I_{\mathrm{MM}}^{\left(n\right)}}{\sum_{k}O_{\mathrm{QM}}^{\left(k\right)}I_{\mathrm{QM}}^{\left(k\right)}O_{\mathrm{MM}}^{\left(k\right)}I_{\mathrm{MM}}^{\left(k\right)}}$$

Note that *σ*^(*n*)^ satisfies the previously mentioned boundary conditions. Then, the effective potential energy *V*^eff^ in MD simulations is

(9) $$V^{\mathrm{eff}}\left(r\right)=\overset{N}{\underset{n}{\sum }}\sigma^{\left(n\right)}\left(r\right)V^{\left(n\right)}\left(r\right)$$

Since the weight function *σ* is normalized, the resulting effective forces and energy are always kept in a physically plausible scale, even if limited number of partitionings are considered, which is one of the advantages of using a size-consistent multipartitioning scheme. Furthermore, because the weight functions are perfectly continuous, the partitioning update does not cause any abrupt changes in the effective energy and forces.

## 3. Modification of the Update Protocol

The MD stability problem arises from the character of the SCMP weight function; the weight functions can become zero in limit of ordered or disordered partitioning. In addition, the number of partitionings to be considered is limited in practice. As a result, it is possible to have only a small number of partitionings with large weights, which would cause problems of stability and spatial continuity.

To better understand the stability problem, consider a solvent molecule that is defined as QM in all the weighted partitionings. If the QM solvent molecules diffuse beyond *t*_QM_ from a QM solute molecule, all the partitionings can have simultaneously a weight of zero, which would result in the collapse of the MD simulation. This would also be the case for the solvent molecules that are defined as MM in all the weighted partitionings. Therefore, to achieve the stability of the MD simulations, the partitionings should have variety in the selection of the QM solvent molecules and as many partitionings as possible should have nonzero weight. To assess simulation stability, let us define *σ*_max_ as

(10) $$\sigma_{\max}=\max\left(\sigma^{\left(1\right)},\sigma^{\left(2\right)},\cdots ,\sigma^{\left(n\right)}\right)$$

Note that *σ*_max_ ranges from 1/*N* to 1, where *N* represents the total number of partitionings. To achieve stable simulations, *σ*_max_ should be kept as small as possible.

Spatial continuity denotes a smooth connection between QM and MM regions. In other words, solvent models are smoothly alternated when solvent molecules cross the QM/MM border. To intuitively understand spatial continuity, in a previous study [11], we introduced a useful measure: the instantaneous QM profile *ω_t_*(*j*)

(11) $$\omega_{t}\left(j\right)=\overset{N}{\underset{n}{\sum }}\sigma_{t}^{\left(n\right)}\delta_{j}^{\left(n\right)}$$

where

(12) $$\delta_{j}^{\left(n\right)}=\{\begin{array}{c} 1\;\mathrm{if}\;j\in S_{\mathrm{QM}}^{\left(n\right)}\\ 0\;\mathrm{otherwise} \end{array}$$

and *ω_t_*(*j*) describes how much a solvent molecule that is the *j*-th nearest neighbor to the QM solute behaves as a QM molecule at time *t* [11]. 

Since *σ*^(*n*)^ is normalized in Equation (8), a QM profile ranges from 0 to 1 where the values of unity or zero indicate that the solvent molecule corresponds to a perfectly QM or MM model, respectively. Thus, as a solvent molecule approaches the QM solute, the QM profile should become larger and vice versa. Therefore, to have a smooth connection between the QM and MM regions, the QM profiles should gradually and monotonically decrease as the molecular number *j* increases. Then, the time-averaged QM profile *ω*(*j*) is written as

(13) $$\omega \left(j\right)=\frac{1}{T}\overset{T}{\underset{t=1}{\sum }}\omega_{t}\left(j\right)$$

Then, we evaluated the standard deviation of the QM profile of the *j*-th solvent molecule as

(14) $$\mathrm{SD}\left(j\right)=\sqrt{\frac{1}{T}\overset{T}{\underset{t=1}{\sum }}{\left(\omega_{t}\left(j\right)-\omega \left(j\right)\right)}^{2}}$$

### 3.1. Partitioning Update Types

In this section, we discuss the SCMP update types and modify the previous update scheme for efficiency. Let a QM inside entry be a process where a partitioning with *σ* = 0, because of either *I*_QM_ = 0 or *I*_MM_ = 0, at a certain MD step becomes effectively weighted at the next MD step. Likewise, let MM inside entry be a process where a partitioning has nonzero weight because *I*_MM_ > 0. Here, we note that inside updates are not always available depending on the range parameters *s* and *t*. For example, suppose all partitionings have consistently *m* QM solvent molecules. Let *d_j_* be the distance between the QM solute and the *j*-th nearest solvent molecule. Note that *I*_QM_ = 0 for *s*_QM_ ≥ *d_m_* and *I*_MM_ = 0 for *d_m_*_+1_ ≥ *t*_MM_. Since *d_j_* fluctuates during the MD simulations, if *t*_MM_ > *s*_QM_, there can be a moment in which *t*_MM_ > *d_m+_*_1_ ≥ *d_m_* > *s*_QM_. In this case, partitionings satisfying either *I*_QM_ = 0 or *I*_MM_ = 0 do not exist. If *s*_QM_ ≥ *t*_MM_, in contrast, there always exists at least one zero-weighted partitioning satisfying either *I*_QM_ = 0 or *I*_MM_ = 0 regardless of *d_m_*. To make the update available at every MD time step, we assume *s*_QM_ ≥ *t*_MM_ hereafter.

Next, let us compare the conditions *s*_QM_ = *t*_MM_ and *s*_QM_ > *t*_MM_. To have stable simulations, ideally, partitionings should have nonzero weights immediately after the update. Suppose that an updated partitioning with *σ* = 0 has *I*_QM_ = 0 for *s*_QM_ > *d_m_*. Because of the diffusion of the QM solvent molecules, sooner or later, the updated partitioning by inside entry will have a nonzero weight, when *s*_QM_ < *d_m_*. However, if *s*_QM_ >> *d_m_*, it would take time for the partitioning to have a nonzero weight; as a result, a small number of partitionings would have large weights, making the simulation unstable. Thus, *s*_QM_ should be as small as possible. For the same reason, *t*_MM_ should be as large as possible. Therefore, we assume that *s*_QM_ = *t*_MM_ is the most efficient situation to suppress *σ*_max_ and this will be the condition that we will apply for efficient update unless otherwise stated.

Let a QM outside entry be a process where a disordered partitioning with *σ* = 0 because *O*_QM_ = 0 happens to be reweighted again. For instance, suppose that a QM solvent molecule diffuses beyond *t*_QM_ and accordingly *O*_QM_ = 0. Then, the diffused QM solvent molecule may happen to come back within *t*_QM_ again, leading to *σ* > 0 with *O*_QM_ > 0. Likewise, let an MM outside entry be a process where *σ* > 0 when an MM solvent molecule moves beyond *s*_MM_ of the QM solute. In contrast to the QM entries, the MM entries are always available regardless of range parameters *s* and *t*.

Note that partitionings updated by any of the four types, in particular MM inside and QM outside entries, may not necessarily be weighted again. For instance, while waiting for *σ* > 0 by a QM outside entry, the MM fade-out functions can become *O*_MM_ = 0. In such case, it is less likely that this partitioning can have a nonzero weight again in the limited simulation time. Thus, partitionings updated should be carefully checked to see if there are better candidates with nonzero weights among other possible partitionings. In contrast, a QM outside entry seems to happen more frequently because the sphere surface at *r = t*_QM_ is larger than those at *r = s*_MM_ and *s*_QM_ and therefore, the frequency of solvent molecules crossing the surface is also higher. 

We also note that, in previous studies, we made only use of the inside entry for partitioning update. In this case, limited partitionings are available for update, which seems to be inefficient, if many partitionings have to be updated at the same time because of partitioning overlap. In the present study, we propose to exploit the outside entries to effectively control *σ*_max_. For efficiency, when a partitioning becomes disordered (*σ* = 0 by either *O*_QM_ = 0 or *O*_MM_ = 0), we make the partitioning partially-ordered by tuning the solvent molecules irrelevant to *O* = 0. Otherwise, as previously mentioned, the outside-entered partitioning is highly likely to become disordered again. 

### 3.2. Degree of Order

Under the condition that the range parameters *s*_QM_ = *t*_MM_, the perfectly ordered partitioning whose QM region consists of the nearest *m* solvent molecules has always zero weight as described in the previous section. Using this property, in a previous study, we employed a minimum update protocol where the partitioning to be updated is always replaced by the perfectly ordered partitioning, namely the QM region consists of the nearest solvent molecules. Note that when the minimum update is once performed, the partitioning update is not available until solvent diffusion occurs to some extent. Otherwise, more than one partitioning can become identical. As a result, idling times can happen between partitioning updates, which can cause an increase of the maximum weight *σ*_max_ and destabilize the MD simulation. Thus, the minimum update protocol is inefficient.

**Scheme 2:**
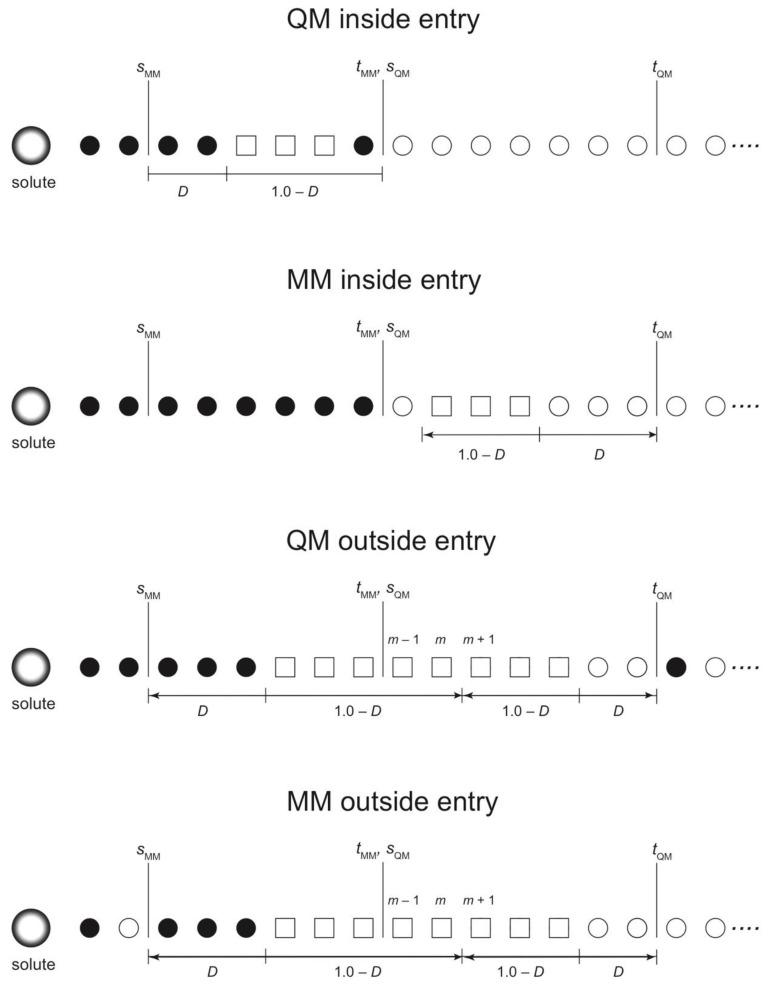
Four possible patterns of partitioning update. Filled and open circles represent QM and MM solvent, respectively. The open squares stand for either QM or MM solvent.

Here, we make the partitioning update more flexible by introducing an index, degree of order (*D*). Note that either *I*_QM_*O*_QM_ or *I*_MM_*O*_MM_ is zero in a new partitioning, while the nonzero one is arbitrary. Thus, keeping *σ* = 0, new partitionings can be disordered to some extent. The degree of order *D* indicates how ordered a new partitioning is. If *D* = 1, the fade-in function *I* = 0 (*O* = 1) and the scenario is equivalent to that of the minimum update. If *D* = 0, *I* = 1 (*O* = 0), which indicates that the partitioning is already completely disordered. The four types of update protocols in combination with the degree of order are visualized in Scheme 2 and detailed in Appendix A. We assume that the optimal value of *D* makes the updated partitionings to become effectively weighted after an entry. Although the optimal value of *D* is not trivial, it should obviously be between *D* = 0 and *D* = 1. Thus, we assessed the optimal value of *D* in the section below.

## 4. Results

### 4.1. MD Performance

**Figure 1:**
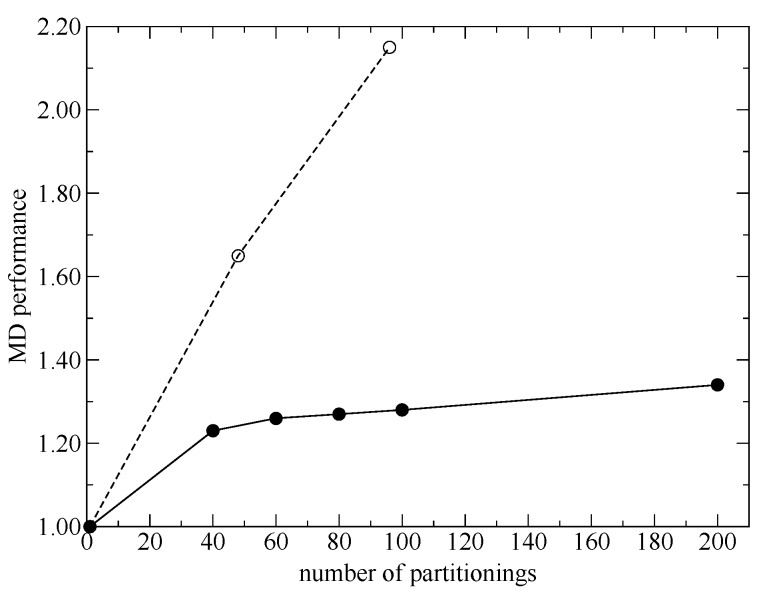
MD performances (wall-time per one MD step) of the SCMP simulations relative to a conventional QM/MM simulation that has the same size of the QM region. Filled circles and the solid line represent the results in the present study and open circles and the dashed line represent the results in a previous study [14]. The benchmark simulations were conducted for a system composed by 2048 water molecules, where the QM region consisted of one solute water and 32 solvent water molecules.

Figure 1 indicates the relative MD performances against the conventional QM/MM simulations. In MD simulations, in particular for QM/MM systems, the computational loads are mainly attributed to the force and energy calculations. The SCMP simulation is designed carried out in parallel and the force and energy calculations for the individual partitionings are assigned to the respective computational nodes. Thus, in parallel computing, the SCMP simulations should theoretically keep high MD performance, namely wall-time per MD step, even when the number of partitionings increases. Nevertheless, in previous studies, MD performance was substantially affected by the number of partitionings [14], which implies that computational loads specific to the SCMP method, namely the weight calculations, are comparable to the force and energy calculations. This agrees with the fact that the linear extrapolation of the SCMP performance does not agree with that of the conventional QM/MM MD simulations, based on a single partitioning. We find that most parts of the weight calculations, that imply the evaluations of the score and fade-in/out functions, are fortunately independent among partitionings and thus they can be also trivially parallelized. 

Furthermore, the fade-out and fade-in functions share the same score functions *Λ* as in Equations (4)–(7). Although it is necessarily required to share the functions, there is no legitimate reason to use different functions. Since the sharing eliminates redundancy, it is advantageous with respect to the computational cost; in addition, the SCMP overview and programming is simplified. Thus, in the following section, we use the same range parameters *s* and *t* for the QM and MM score functions. 

To assess MD performance in the weight calculations via parallelization and parameter sharing, we conducted benchmark simulations on a computer server with an Intel Xeon E5-2670v2 CPU. As shown in Figure 1, the present study successfully elicited the potential efficiency of the SMCP method and showed high MD performance and robustness with respect to the number of partitionings. Note that the MD performance shows slightly linear dependency on the number of partitionings, which seems to be originated from the partitioning update in the SCMP simulation. Thus, room may still remain for further improvement, although its parallelization is not as straightforward as that of the weight calculation.

### 4.2. Simulation Stability

**Figure 2:**
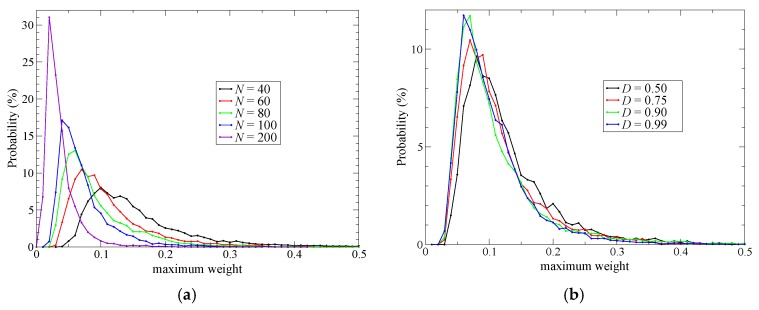
Distribution of the maximum value, *σ*_max_(*t*), sampled over 100 ps MD simulations where respective partitionings contain 1 QM solute and 32 QM solvent molecules. (**a**) The black, red, green, blue and purple lines represent the simulations with 40, 60, 80, 100 and 200 partitionings, respectively. All the simulations employed *D* = 0.75; (**b**) The black, red, green and blue lines represent the results from the simulations with *D* = 0.50, 0.75, 0.90 and 0.99, respectively; all the simulations were performed with 60 partitionings.

To evaluate simulations stability, we conducted SCMP simulations with different numbers of partitionings (40, 60, 80, 100 and 200) and evaluated the distribution of *σ*_max_ sampled along the MD simulations as shown in Figure 2a. As expected, the maximum value of the partitioning weights obviously depends on the number of partitionings; a larger number of partitionings leads to a smaller *σ*_max_ value. 

Next, we evaluated the distribution of *σ*_max_ for a SCMP simulation with 60 partitionings using various degrees of order (*D* = 0.50, 0.75, 0.90 and 0.99) as shown in Figure 2b. Notably, a larger value of *D* seems to keep *σ*_max_ smaller, although there is not a distinct difference for *D* = 0.90 and 0.99. Thus, we conclude that in what regards simulation stability, the optimal value of *D* seems to be between 0.90 and 0.99. On the contrary, we emphasize that all the simulations conducted in the present study lasted for 100 ps and never collapsed even with an unfavorable condition such as *D* = 0.50. This is in contrast to previous studies where the MD simulations sometimes crashed. We think that stabilization is achieved because of the new partitioning update protocol. While we used only two protocols (QM and MM inside entries) in previous studies, we made use of four types of the partitioning update protocol in the preset study (see Appendix A).

### 4.3. Spatial Continuity

**Figure 3:**
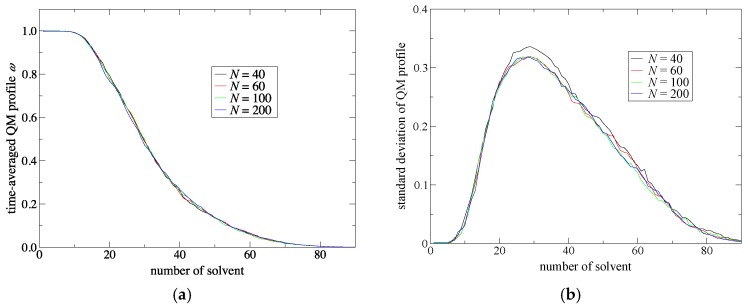
(**a**) Time-averaged QM profiles, *ω*, and (**b**) their standard deviations obtained from 100 ps SCMP simulations. The horizontal axis represents the solvent number from the QM solute. Black, red, green and blue lines represent the results obtained by the SCMP method with number of partitionings equal to 40, 60, 100 and 200, respectively.

To assess the spatial continuity, we compared the QM profiles in Equations (11) and (13) employing different number partitionings (40, 80, 100 and 200). The QM profiles are subject to the score function *Λ* in Equation (1) through Equations (4)–(9), although it is not straightforward to guess the resulting form of the QM profile from the score function. As shown in Figure 3a, the time-averaged QM profiles over the MD simulations smoothly and monotonically decrease as the distance from the QM center increases. In addition, they are not affected by the number of partitionings at least in the range from 40 to 200 partitionings. Therefore, spatial continuity seems to be achieved with respect to time average. Note that this does not necessarily ensure instantaneous spatial continuity, which is defined at every MD time step and estimated by instantaneous QM profile.

To discuss instantaneous spatial continuity, we evaluated the standard deviations (SDs) of the instantaneous QM profiles along the MD simulation as shown in Equation (14). If the instantaneous QM profiles agree with the time averaged QM profiles at every MD time step, the standard deviation should be small. However, Figure 3b shows that the obtained SDs are remarkably large, with a maximum value of about 0.3 at around the 32nd solvent molecule from the QM solute. It is also notable that the solvent number, 32nd, showing the largest SD value agrees with the number of QM solvent molecules contained in the respective partitionings. These large SDs imply that the QM profiles significantly fluctuate in the course of the MD time. As a result, the entire shape of the instantaneous QM profiles does not agree with the expected shape (time-averaged QM profiles) displayed in Figure 3a. In other words, there is still a room for improvement of spatial continuity for MD time step, while it can be achieved with respect to time average.

We emphasize that, if all possible size-consistent partitionings are considered, perfect instantaneous spatial continuity should be achieved. Therefore, as the number of partitionings increases, the SDs of the QM profiles should be reduced. Notably, however, Figure 3b shows that the increase in the number of partitionings in the range from 60 to 200 does not cause the decrease of the associated SD, although the SDs corresponding to the QM profile obtained from the SCMP simulation carried out with 40 partitionings is the largest.

**Figure 4:**
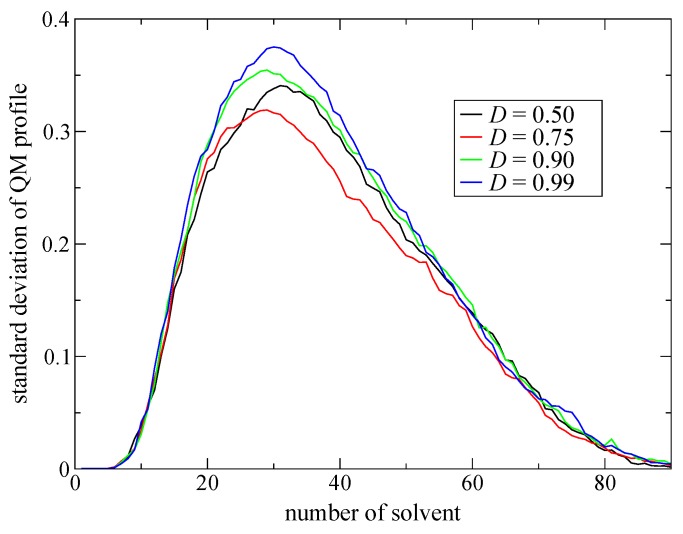
Standard deviations of instantaneous QM profiles, *ω_t_*, obtained from 100 ps SCMP simulations with 60 partitionings containing one QM solute water and 32 QM solvent water molecules. The horizontal axes represent the solvent number from the QM solute. Black, red, green and blue lines represent the results obtained by SCMP with degrees of order *D* = 0.50, 0.75, 0.90 and 0.99, respectively.

Next, we assessed the SDs of the QM profiles obtained using 60 partitionings and different degrees of order *D* = 0.50, 0.75, 0.90 and 0.99. Figure 4 indicates that SDs vary depending on *D*; *D* = 0.75 gives the smallest SD value, namely the best spatial continuity. Notably, the values of *D* that lead to the best spatial continuity and the best simulations stability are not the same. 

**Figure 5:**
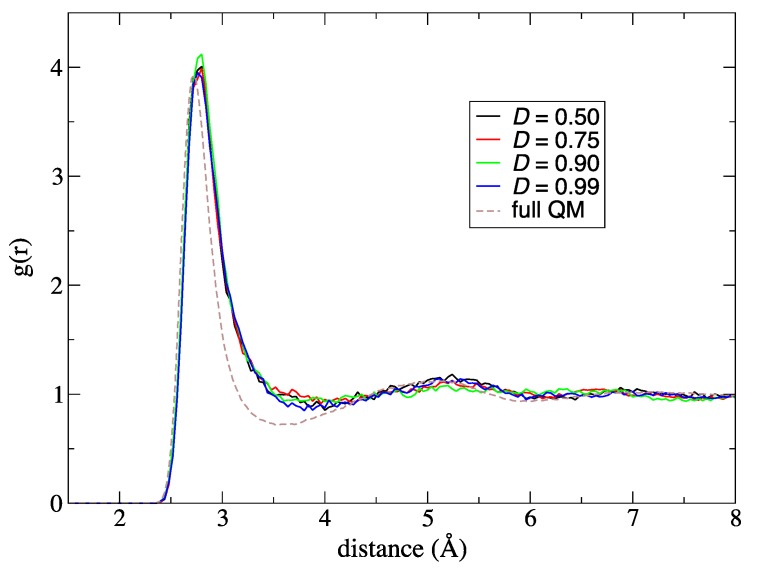
RDFs obtained from 100 ps SCMP simulations with 60 partitionings. Black, red, green and blue lines represent the results obtained by SCMP with degrees of order *D* = 0.50, 0.75, 0.90 and 0.99, respectively. Green line represents the result obtained by the previous SCMP code. Dashed line stands for the full DFTB3/3OB result.

To evaluate the effect of spatial continuity on solvation structure, we calculated the time-averaged QM solute oxygen-centered radial distribution functions for the solvent oxygen atoms (Figure 5). Notably, the radial distribution functions (RDFs) do not show any distinct difference for different values of *D*. We also did not observe any clear difference in the RDFs when compared to different number of partitionings (data not shown). Considering that all the present simulations realize smooth switching of the QM profiles on time-average (Figure 3a) and that the present RDFs are also time-averaged, it seems reasonable that the RDFs correspond to the time-averaged QM profiles (and not with the instantaneous QM profiles). Since the smooth QM profiles on time average were already achieved even with previous code [11], it is also reasonable that any difference does not appear in the RDFs. When compared full DFTB3/3OB results, the SCMP reproduced the first and second peaks of RDF well, while the first minimum is shifted to larger distance.

In a previous study, we showed that the dynamics of a single QM solute surrounded by MM solvent molecules, which is an extreme case of spatial discontinuity, is neither equivalent to that of a pure QM system nor to that of an MM one. Thus, if the QM and MM regions are directly connected as in most of the single-partitioning QM/MM methods, the solvent molecules around the QM/MM border can show unphysical behaviors. In contrast, the gradual transition of solvent molecules between QM and MM is not physically legitimate but it is an implicitly required condition in multipartitioning methods. Indeed, some multipartitioning methods such as PAP, SAP and DAS are designed to perfectly achieve spatial continuity at every MD time step and Park et al., demonstrated by using the DAS method that the location of the transition region can affect the resulting physical quantity [15], although this effect cannot be attributed only to spatial continuity problems because SAP and DAS cannot deal with temporal discontinuity problems. Therefore, it still remains an open question which connection can yield more reasonable results. Although we did not confirm any effect of instantaneous spatial continuity in the present study, at least within the SCMP framework, it is preferable to have a QM profile decreasing monotonically to suppress artifacts around the QM center because of the QM/MM border. 

## 5. Computational Details

All the calculations were carried out with a local version of the GROMACS 5.0.7 package [16,17,18,19] where we implemented the DFTB3 code provided by Dr. Tomáš Kubař [20] and our own SCMP code. We employed DFTB3 [21] with 3OB parameters [22] to treat the molecules in the QM part and the SPC-Fw model [23] to describe MM water molecules. The electrostatic interactions within the MM part were evaluated using the particle-mesh Ewald method [24] with a switching function from 8.5 to 9.0 Å. The MD simulations were conducted under periodic boundary conditions in the *NVE* ensemble, with a time step of 0.5 fs and after inserting 2048 H_2_O molecules in a cubic box with a side length of 39.48 Å. The range parameters were set as *s*_MM_ = 3.5 Å and *t*_MM_ = *s*_QM_ = 6.0 Å and *t*_QM_ = 8.8 Å.

## 6. Conclusions

The SCMP method enables to incorporate quantum chemical solvent effects in MD simulations. First, in the present study, we proposed a simple form for the score functions used in the weight evaluations and discussed an additional parallelization scheme. Then, we benchmarked the MD performance and demonstrated that the SCMP potential efficiency was well elicited even for highly parallel computations.

Next, to improve simulation stability and spatial continuity, we proposed new protocols for the partitioning update and introduced an index, degree of order. We focused on the maximum weight at every MD time step as a measure of the stability of the simulations. Then, we found an extended partitioning update protocol that increases the simulation stability; as a result, we did not face any collapse in the MD simulation performed in the present study. We also demonstrated that the number of partitionings is directly connected to the simulation stability. In addition to the extended update protocol, we introduced the degree of order, which affects simulation stability to some extent too. Benchmark simulations showed that there is an optimal value for the degree of order, which presumably depends on the simulation condition. For spatial continuity, we used the QM profile as an index and demonstrated that the present simulations achieved spatial continuity on time average over MD simulations regardless of the number of partitionings (at least in a range from 40 to 200). Notably, however, instantaneous spatial continuity at every MD time step was not necessarily satisfactory containing large errors. While the number of partitionings, which saturates around 60 partitionings, is not crucial to achieve instantaneous spatial continuity, the degree of order in the partitioning update protocol seems to be influential. Concerning the degree of order, notably, the optimal values to achieve spatial continuity and simulation stability do not necessarily match. Because RDFs are also obtained by time-averaged analysis, we could not observe any distinct difference in RDFs in what regards to instantaneous spatial continuity. There still remains room for further research on evaluating spatial continuity.

## Appendix A

**QM inside entry**: Among the solvent molecules within *s*_QM_ of the QM solute, set the furthest solvent molecule QM. Let *k* solvent molecules be in the range from *s*_MM_ to *t*_MM_ (*s*_QM_) from the QM center. The nearest *D* × *k* solvent molecules are defined as QM, while the rest of *k* solvent molecules are defined as either QM or MM molecules in a new partitioning.

**MM inside entry**: Among the solvent molecules more than *t*_MM_ from the QM solute, set the nearest solvent molecule MM, while the solvent within *t*_MM_ of the QM solute should be QM. Let *k* solvent molecules be in the range from *t*_MM_ (*s*_QM_) to *t*_QM_ from the QM center. The furthest (1 – *D*) × *k* solvent molecules are defined as MM, while the rest of *k* solvent molecules are defined as either QM or MM molecules in a new partitioning.

**QM outside entry**: Among the solvent molecules more than *t*_QM_ from the QM solute, the nearest one should be QM. Suppose the QM region contains *m* QM solvent molecules and the *m*-th solvent is at a distance larger than *r_m_* from the QM center. Let *k* solvent molecules ranging from *r_m_* to *t*_QM_ from the QM center. The furthest *D* × *k* solvents are defined as MM. The rest of the *k* solvents are defined as either QM or MM. Likewise, let be *l* solvent molecules ranging from *r_m_* to *t*_QM_ from the QM center. The nearest *D* × *l* solvent molecules are defined as QM. The rest of the *l* solvent molecules are defined as either QM or MM.

**MM outside entry**: Among the solvent molecules within *s*_MM_ of the QM solute, the furthest one should be MM. Let the QM region contain *m* QM solvent molecules and *r_m_* be the distance between the *m*-th solvent molecule and the QM center. Suppose *k* solvent molecules exist in the range from *r_m_* to *t*_QM_. The furthest *D* × *k* solvent molecules are defined as MM. The rest of the *k* solvent molecules are defined as either QM or MM. Likewise, let *l* solvent molecules exist in the range from *r_m_* to *t*_QM_ from the QM center. The nearest *D* × *l* solvent molecules are defined as QM. The rest of the *l* solvent molecules are defined as either QM or MM.
